# Hepatic Senescence Accompanies the Development of NAFLD in Non-Aged Mice Independently of Obesity

**DOI:** 10.3390/ijms22073446

**Published:** 2021-03-26

**Authors:** Ioannis I. Moustakas, Angeliki Katsarou, Aigli-Ioanna Legaki, Iryna Pyrina, Konstantinos Ntostoglou, Alkistis-Maria Papatheodoridi, Bettina Gercken, Ioannis S. Pateras, Vassilis G. Gorgoulis, Michael Koutsilieris, Triantafyllos Chavakis, Antonios Chatzigeorgiou

**Affiliations:** 1 Department of Experimental Physiology, Medical School, National and Kapodistrian University of Athens, 11527 Athens, Greece; ioamoustakas@med.uoa.gr (I.I.M.); angelikikat@med.uoa.gr (A.K.); elinalegaki@med.uoa.gr (A.-I.L.); alkistispapath@gmail.com (A.-M.P.); mkoutsil@med.uoa.gr (M.K.); 2 Institute for Clinical Chemistry and Laboratory Medicine, University Clinic Carl Gustav Carus, Technische Universität Dresden, 01307 Dresden, Germany; Iryna.Pyrina@uniklinikum-dresden.de (I.P.); Bettina.Gercken@uniklinikum-dresden.de (B.G.); Triantafyllos.Chavakis@uniklinikum-dresden.de (T.C.); 3 Molecular Carcinogenesis Group, Department of Histology and Embryology, School of Medicine, National and Kapodistrian University of Athens, 11527 Athens, Greece; kostasntostoglou@gmail.com (K.N.); ipateras@med.uoa.gr (I.S.P.); vgorg@med.uoa.gr (V.G.G.); 4 Biomedical Research Foundation, Academy of Athens, 11527 Athens, Greece; 5 Molecular and Clinical Cancer Sciences, Manchester Cancer Research Centre, Manchester Academic Health Sciences Centre, University of Manchester, Manchester M20 4GJ, UK; 6 Center for New Biotechnologies and Precision Medicine, Medical School, National and Kapodistrian University of Athens, 11527 Athens, Greece

**Keywords:** NAFLD, age, hepatocyte, replicative senescence, stress-induced senescence, obesity

## Abstract

Senescence is considered to be a cardinal player in several chronic inflammatory and metabolic pathologies. The two dominant mechanisms of senescence include replicative senescence, predominantly depending on age-induced telomere shortening, and stress-induced senescence, triggered by external or intracellular harmful stimuli. Recent data indicate that hepatocyte senescence is involved in the development of nonalcoholic fatty liver disease (NAFLD). However, previous studies have mainly focused on age-related senescence during NAFLD, in the presence or absence of obesity, while information about whether the phenomenon is characterized by replicative or stress-induced senescence, especially in non-aged organisms, is scarce. Herein, we subjected young mice to two different diet-induced NAFLD models which differed in the presence of obesity. In both models, liver fat accumulation and increased hepatic mRNA expression of steatosis-related genes were accompanied by hepatic senescence, indicated by the increased expression of senescence-associated genes and the presence of a robust hybrid histo-/immunochemical senescence-specific staining in the liver. Surprisingly, telomere length and global DNA methylation did not differ between the steatotic and the control livers, while malondialdehyde, a marker of oxidative stress, was upregulated in the mouse NAFLD livers. These findings suggest that senescence accompanies NAFLD emergence, even in non-aged organisms, and highlight the role of stress-induced senescence during steatosis development independently of obesity.

## 1. Introduction

Senescence is a state of cell cycle arrest and inability of the cell to proliferate, while at the same time, senescent cells are resistant to apoptotic cell death [1,2]. During senescence, cells acquire a senescence-associated secretory phenotype (SASP), namely a molecular signature characterized by the secretion of cytokines and chemokines, including interleukin-6 (IL-6) and monocyte chemoattractant protein-1 (MCP-1), as well as a plethora of growth factors and metalloproteinases (MMPs). SASP-related mediators affect the tissue microenvironment by attracting immune cells that are capable of removing senescent cells, whilst simultaneously acting as paracrine inducers of senescence in neighboring cells [1,3,4]. Two main mechanisms of senescence are known, namely replicative and stress-induced senescence. The former is related to aging and depends on telomere length reduction in cells which have undergone a limited number of divisions, while stress-induced senescence is mediated by intracellular or external harmful stimuli, leading to DNA damage [2,5]. Senescence is thought to participate as a cause or consequence in several inflammatory and metabolic disorders, including obesity, metabolic syndrome, and type 2 diabetes [4,6].

Nonalcoholic fatty liver disease (NAFLD) is characterized by fat deposition in hepatocytes, defined as steatosis. This condition is considered to be the demonstration of metabolic syndrome (MS) in the liver, as it is associated with insulin resistance, dyslipidemia, and hypertension, especially in type 2 diabetic patients. Visceral obesity and increased body mass index (BMI) are major risk factors for the development of NAFLD [7,8]. Additionally, NAFLD and MS are associated in a bidirectional manner, since excess lipid content in the liver is able to provoke a low-grade inflammatory process, promoting hepatic insulin resistance as well as other pathologies such as dysregulated lipid metabolism and atherosclerosis [9,10]. Importantly, NAFLD may progress to nonalcoholic steatohepatitis (NASH), a condition featuring inflammation and fibrosis, in addition to steatosis; NASH can further evolve into cirrhosis or hepatocellular carcinoma [11,12,13]. NAFLD prevalence increases with age and is thought to affect males in higher proportions compared to females. Specifically, according to the National Health and Nutrition Examination Surveys (NHANES-III) study, NAFLD prevalence in men peaks between 51 and 60 years of age, while it peaks in women above 60 years of age, with a distribution of 29.3% and 25.4%, respectively. Nonetheless, 16.1% of males and 12.5% of females are presented with NAFLD between the ages of 30 and 40, implying that although age increases the risk for NAFLD, other pathogenic mechanisms, especially adiposity, are also of major importance for the emergence of the disease [14,15].

Senescence has been implicated in the development of liver steatosis during NAFLD, and the genetic or pharmacological elimination of senescent hepatocytes significantly reduced fat accumulation in mouse livers [16]. Previous studies in both rodents and humans have studied the effect of obesity and related metabolic dysregulation on the development of senescence in hepatocytes [16,17,18]. Nevertheless, the majority of studies in mice have focused on age-induced senescence during NAFLD. Moreover, in humans, telomere length, an indicator of replicative senescence, is predominantly used to study senescence in the context of NAFLD, while stress-induced senescence is poorly studied [16,18,19,20]. A plethora of mechanisms, such as fatty acid abundance in the liver microenvironment, mitochondrial dysfunction, or hepatic inflammation, can provoke hepatic senescence contributing to NAFLD [21,22]. The question of whether these NAFLD-triggering mechanisms are linked to replicative or/and stress-induced senescence in non-aged organisms, in the presence or absence of obesity, remains unanswered. Furthermore, it is not clear whether or not replicative senescence and stress-induced senescence co-exist in NAFLD of non-aged organisms, which represents the core question addressed in the current study.

## 2. Results

### 2.1. Animal Models of NAFLD in Non-Aged Mice

**Figure 1:**
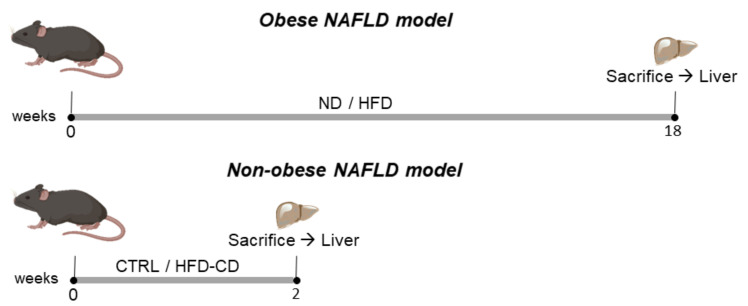
Animal models of nonalcoholic fatty liver disease (NAFLD) in non-aged mice. Eight-week-old C57Bl/6 mice were subjected to a long- and short-term model of diet-induced NAFLD. For the long-term obese model, the mice were fed a normal-fat diet or a high-fat diet (ND or HFD with 10% and 60% of kcal from fat, respectively) for 18 weeks, while for the short-term non-obese model, the mice were fed a high-fat, choline-deficient, low-methionine diet (HFD-CD) or a standard control diet (CTRL) for 2 weeks.

We engaged two NAFLD models in non-aged mice. Specifically, young adult mice were subjected to two different dietary models of NAFLD which differed in the presence of obesity. The first NAFLD model was based on diet-induced obesity resulting from feeding on a high-fat diet (HFD) for 18 weeks. In the second, non-obese NAFLD model, mice were fed a high-fat, choline-deficient, low-methionine diet (HFD-CD) for 2 weeks [23,24] (Figure 1).

**Figure 2:**
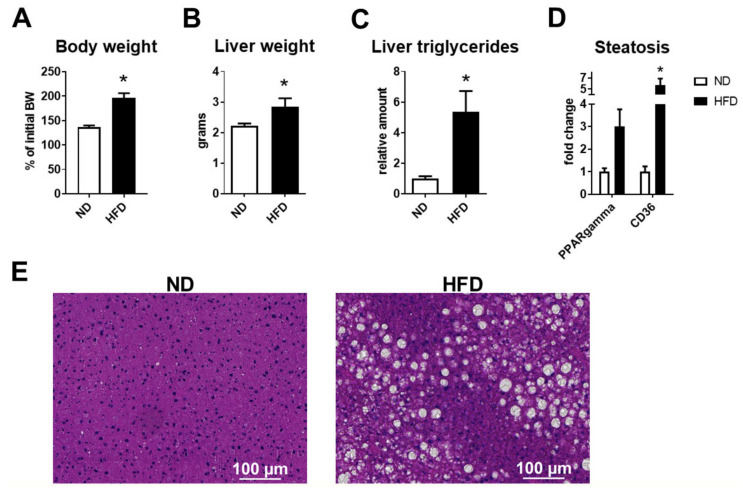
Development of liver steatosis in the model of HFD-induced NAFLD. (**A**) Body weight of mice fed an ND or an HFD is shown. Data are presented as percentages of the initial body weight. (**B**) Liver weight of ND- and HFD-fed mice is shown. (**C**) The triglyceride levels in hepatic tissue homogenates from ND- and HFD-fed mice were quantified. The triglyceride amounts (μmol of triglycerides per gram of tissue) are expressed relative to those of ND mice, which were set as 1. (**D**) The mRNA expression of steatosis-related genes (peroxisome proliferator-activated receptor gamma, PPAR-gamma, CD36) in livers from ND- and HFD-fed mice was evaluated by qPCR. Eukaryotic translation elongation factor 2 (ETEF2) was used for normalization of mRNA expression, and the expression of each gene in the control group (ND) was set as 1. (**E**) Representative images of hematoxylin–eosin staining in liver sections of ND- or HFD-fed mice. Data are presented as mean ± SEM (*n* = 8/group); * *p* < 0.05.

**Figure 3:**
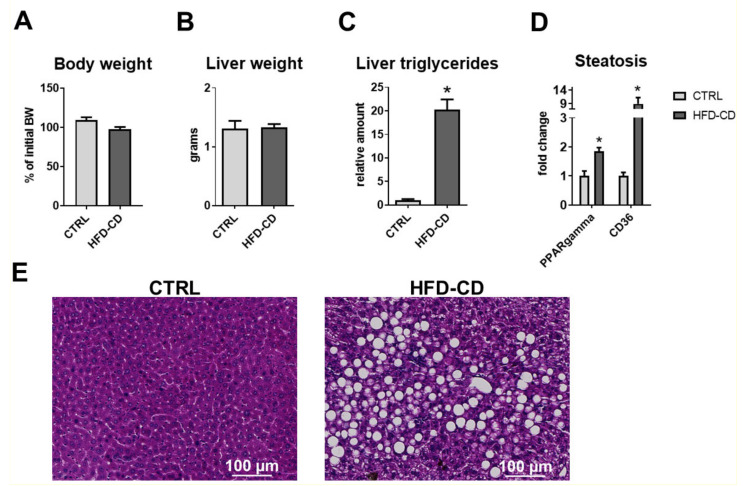
Development of liver steatosis in the model of HFD-CD-induced NAFLD. (**A**) Body weight of mice fed a CTRL or an HFD-CD diet is shown. Data are presented as percentages of the initial body weight. (**B**) Liver weight of CTRL- and HFD-CD-fed mice. (**C**) The triglyceride levels in hepatic tissue homogenate from CTRL- and HFD-CD-fed mice were measured. The triglyceride amounts (μmol of triglycerides per gram of tissue) are expressed relative to those of CTRL mice, which were set as 1. (**D**) The mRNA expression of genes related to hepatic fat accumulation (PPAR-gamma, CD36) in livers from CTRL and HFD-CD mice was evaluated by qPCR. ETEF2 was used for normalization of mRNA expression, and the expression of each gene in the control group (CTRL) was set as 1. (**E**) Representative images of hematoxylin–eosin staining in livers from CTRL- and HFD-CD-fed mice. Data are presented as mean ± SEM (*n* = 4–5/group); * *p* < 0.05.

The obese HFD-fed mice displayed significantly increased body and liver weight as compared to the group that was fed a normal diet (ND) (Figure 2A,B), accompanied by increased levels of tissue triglycerides and enhanced mRNA expression of lipid accumulation-related genes such as peroxisome proliferator-activated receptor gamma (PPAR-gamma) and CD36 in the liver (Figure 2C,D). Steatosis was confirmed by histological examination of hematoxylin and eosin (H&E)-stained liver sections, shown in Figure 2E. In contrast, no difference in body weight or liver weight was observed in the mice fed the HFD-CD diet for 2 weeks, as compared to their respective control mice; see Figure 3A,B. Nevertheless, extensive steatosis was observed in the HFD-CD group, characterized by a robust upregulation of hepatic triglyceride levels, increased expression of steatosis- and lipid uptake-related genes, as well as the histological presence of hepatic fat deposition (Figure 3C–E).

### 2.2. Senescence Accompanied Steatosis during NAFLD Development

**Figure 4:**
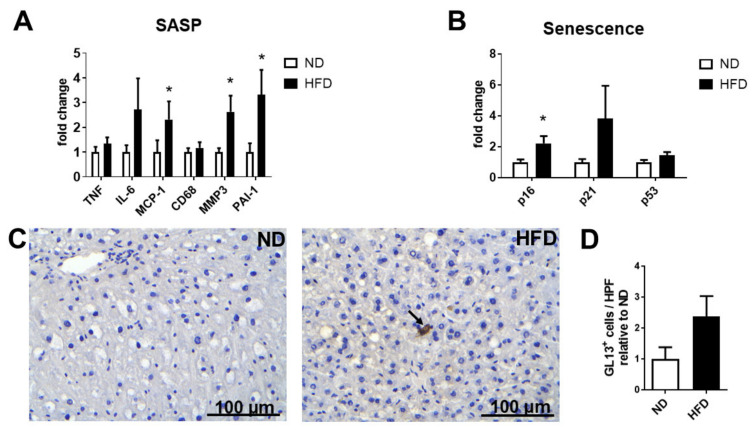
Senescence in the livers of mice of the HFD-induced model of NAFLD. (**A**,**B**) The mRNA expression of senescence-associated secretory phenotype (SASP)-related genes (**A**) or senescence-related genes (**B**) was assessed by qPCR in livers from ND- and HFD-fed mice. ETEF2 was used for normalization of mRNA expression, and the expression of each gene in the ND group was set as 1. (**C**) Representative images of GL13 staining indicating senescence in livers of ND- and HFD-fed mice are shown. Arrow indicates a GL13-positive cell. (**D**) The number of GL13-positive cells per high-power field (HPF), as shown in (**C**), was evaluated and data are displayed as relative to the cells/HPF of the ND group. Data are presented as mean ± SEM (*n* = 7–8/group); * *p* < 0.05.

Hepatic senescence was investigated in both models of diet-induced NAFLD. The gene expression of several SASP component molecules, such as those of monocyte chemoattractant protein-1 (MCP-1), matrix metalloproteinase 3 (MMP3), and Plasminogen activator inhibitor-1 (PAI-1), was upregulated in the HFD mice as compared to the ND controls, as seen in Figure 4A. Similarly, the gene expression of the senescent regulatory genes p16, p21, and p53 was upregulated in the HFD group; however, only the increased expression of p16 reached significance (Figure 4B). Along this line, an increased presence of senescent cells, defined as GL13-positive cells, was shown in the livers of the HFD-fed mice as compared to ND-fed ones; see Figure 4C,D.

**Figure 5:**
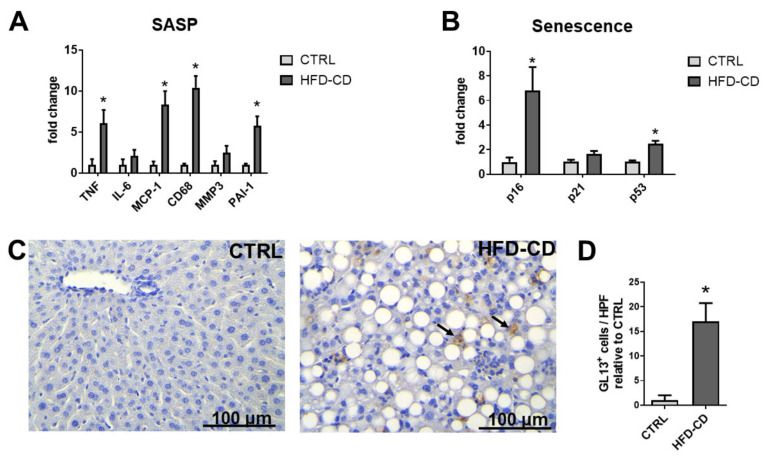
Senescence in the livers of mice of the HFD-CD-induced model of NAFLD. (**A**,**B**) The mRNA expression of SASP-related genes (**A**) or senescence-related genes (**B**) was assessed by qPCR in livers from CTRL- and HFD-CD-fed mice. ETEF2 was used for normalization of mRNA expression, and the expression of each gene in the CTRL group was set as 1. (**C**) Representative images of GL13 staining in livers of CTRL- and HFD-CD-fed mice are shown. Arrows indicate GL13-positive cells. (**D**) The number of GL13-positive cells per high-power field (HPF), as shown in (**C**), was evaluated and data are displayed as relative to the cells/HPF of the CTRL group. Data are presented as mean ± SEM (*n* = 3–5/group); * *p* < 0.05.

In the non-obese HFD-CD feeding model, several SASP-related genes, including genes encoding for Tumor Necrosis Factor (TNF), MCP-1, CD68, and PAI-1, as well as the senescence-related genes p16 and p53 displayed increased mRNA expression in the livers of the HFD-CD group as compared to control mice; see Figure 5A,B. Consistently, immunohistochemistry for GL13 showed that senescent cells in the livers of the HFD-CD group were much more abundant compared to those of the respective control group (Figure 5C,D).

### 2.3. NAFLD of Non-Aged Mice Is Characterized by Stress-Induced Senescence

**Figure 6:**
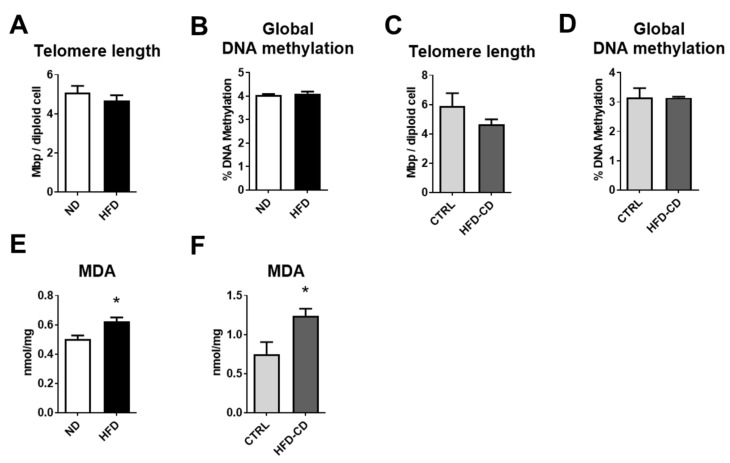
NAFLD in non-aged mice is characterized by stress-induced senescence. (**A**) Total telomere length per diploid cell was evaluated in hepatic tissue of ND- and HFD-fed mice as described in the experimental procedures. (**B**) The percentage of global DNA methylation in DNA isolated from livers of ND and HFD mice is shown. (**C**) Total telomere length per diploid cell was evaluated in livers from CTRL and HFD-CD mice. (**D**) The percentage of global DNA methylation in DNA isolated from livers of CTRL and HFD-CD mice is shown. (**E**,**F**) The amount of malondialdehyde (MDA), as an indicator of oxidative stress, in the livers from ND and HFD mice (shown in **E**) or CTRL and HFD-CD mice (shown in **F**) is shown. In (**A**,**B**,**E**), *n* = 7–8/group; in (**C**,**D**,**F**), *n* = 4–5/group. Data are mean ± SEM; * *p* < 0.05.

Premature replicative senescence is characterized by reductions in telomere length, while alterations in global DNA methylation were previously shown to occur in an age- and nutrition-dependent manner [25]. Neither telomere length nor global DNA methylation were altered between the groups of both NAFLD models, indicating that NAFLD was not associated with replicative senescence in this context; see Figure 6A–D. The other mechanism of senescence, stress-induced senescence, is triggered by reactive oxygen species (ROS), and oxidative stress has a pivotal role in the pathogenesis of NAFLD [26]. The levels of malondialdehyde (MDA), as an indicator of oxidative stress, were measured in the livers of mice from both models. Interestingly, in both models, the levels of MDA were upregulated in the NAFLD livers (Figure 6E,F), implying that NAFLD in non-aged mice may be predominantly linked to stress-induced senescence.

## 3. Discussion

In our present study, senescence accompanied the development of NAFLD in both models of non-aged mice. Although our models differed significantly in the duration of feeding and obesity development, both models displayed extended hepatic steatosis, as revealed by a histological examination and the determination of triglycerides in the liver. The key difference between the two feeding programs was that, although both resulted in liver steatosis, only the HFD caused obesity in mice, while the HFD-CD feeding did not affect body or liver weight. Of note, at the end of both feedings, both groups of mice were still non-aged, as mice less than 8 months old are considered to be young, mature adults, while mice between the ages of 10 and 14 months are characterized as middle-aged [27]. So far, the majority of studies which specifically focused on NAFLD-related senescence have used either genetic models of obesity or presented findings in middle- or old-aged diet-induced obese mice [16,28,29]. Only Zhang et al. used young animals, but their observations were in rats and not in mice [17,30]. Furthermore, our study is the first to use a short-term dietary model of NAFLD, based on HFD-CD, in parallel to the classic high-fat diet.

Our findings reveal, for the first time, that hepatic senescence can occur independently of age and accompanies liver steatosis even in the absence of obesity. Indeed, gene expression analysis as well as a robust senescence-specific staining confirmed the presence of senescence in our models. Importantly, the NAFLD-related senescence was likely due to stress-induced, rather than premature replicative, senescence, since neither the telomere length nor the global DNA methylation differed significantly in both NAFLD models, while MDA levels were upregulated in both models. Changes in telomere length and global DNA methylation are considered important regulators and markers of age-induced senescence in several tissues [2,25]. In humans, several studies implicate telomere shortening in NAFLD and indicate it as a potential marker or therapeutic target, although its role as a disease cause has not yet been proven [31]. A study in mice suggests that telomeropathies and short telomeres can cause metabolic dysfunction in hepatocytes [32]. The absence of considerable change in telomere length in our models indicates that telomere shortening is not involved in NAFLD development in the models used herein, implying that the emergence of NAFLD in non-aged organisms does not correlate with changes in telomere length. In NAFLD, data from both mouse and human studies propose an inverse correlation between global DNA methylation and disease progression [33,34]. In this context, changes in the methylation status of specific steatosis-related genes are considered of importance [35]. Determination of the methylation status of specific NAFLD-related genes was beyond the focus of our study, and thus, we cannot exclude the involvement of such alterations in our models. In contrast, we observed enhanced oxidative stress, as demonstrated by increased hepatic MDA levels, a fact that is consistent with multiple studies highlighting the role of ROS in NAFLD [13,36]. The upregulation of ROS in our NAFLD models could also explain the emergence of stress-induced senescence in both.

In our study, a comprehensive senescence analysis employing qPCR expression analysis of SASP and senescence-related genes, along with a robust hybrid histo-/immunochemical senescence-specific staining, was conducted. The utilization of two distinct diet-induced NAFLD mouse models, differing in the presence of obesity, and the simultaneous analysis of the two major mechanisms of senescence, namely replicative and stress-induced senescence, represent the strengths of our study. On the other hand, using male mice in both of our models represents a limitation of our study. Nonetheless, controversies still exist in the literature regarding the susceptibility of male versus female mice to NAFLD when different diet-induced models of NAFLD are engaged, implying that further research needs to be conducted addressing this issue [37,38].

Overall, our findings are consistent with data from human studies demonstrating that several stimuli, including fatty acid abundance in the liver microenvironment, chronic inflammation, and mitochondrial dysfunction, may provoke NAFLD independent of obesity and age [21,22]. Further research in both mice and humans is required to investigate the effect of different stimuli on the development of NAFLD, in the presence or absence of obesity and among different age categories. Notwithstanding the underlying pathomechanisms triggering NAFLD, senescence may be a common denominator in NAFLD pathogenesis. Hence, interventions which target and delay the emergence of hepatic senescence should be considered as a therapeutic strategy against the disease in the years to come. In accordance, future research approaches should also focus on the effect of pharmacological agents and/or lifestyle interventions, such as caloric restriction and exercise, on NAFLD-related senescence.

## 4. Materials and Methods

### 4.1. Animal Studies

For the induction of nonalcoholic fatty liver disease (NAFLD), 8-week-old C57Bl/6 male mice (Janvier) were subjected to two different models of diet-induced NAFLD. For the long-term obese model, mice were fed a high-fat diet (HFD) with 60% kilocalories from fat, 20% kilocalories from protein, and 20% kilocalories from carbohydrate or a normal diet (ND) with 10% kilocalories from fat (D12492 and D12450B, respectively, Research Diets, New Brunswick, NJ, USA) for 18 weeks [39,40]. For the short-term non-obese model, mice were fed a high-fat, choline-deficient, low-methionine diet (HFD-CD, A06071302, from Research Diets) with 62% kilocalories from fat, 18% kilocalories from protein, and 20% kilocalories from carbohydrate or a standard control diet (CTRL) (V1534-300, Ssniff Spezialdiäten GmbH, Soest, Germany) for 2 weeks [23,24]. Mice were fed ad libitum with free access to water. The mice were euthanized at the end of the feeding periods and their livers were collected. The animal work was approved by the Landesdirektion Sachsen, Germany.

### 4.2. Measurement of Liver Triglycerides

For the quantification of the hepatic triglyceride content, liver tissue pieces were weighed and homogenized in a 5% Triton X-100 buffer. The homogenates were subjected to two cycles of heating at 95 °C and re-cooling at room temperature. Upon centrifugation of the homogenates, the amount of triglycerides in the supernatants was quantified using a commercially available kit (Triglyceride Quantification Assay, Abcam, Cambridge, UK) [41].

### 4.3. RNA Isolation and qPCR

Total RNA from liver tissues was extracted using TRIzol. The RNA concentrations were quantified and subjected to cDNA synthesis by using the PrimeScript™ RT Reagent Kit (Takara, Shiga, Japan). The SsoFast EvaGreen Supermix (BioRad, Hercules, CA, USA) was used to perform qPCR on an iQ5 Bio-Rad cycler system. The calculation of the relative mRNA expression was performed according to the ΔΔCt method [42] by using the expression of eukaryotic translation elongation factor 2 (ETEF2) for normalization among samples [43,44].

### 4.4. Histology and Immunohistochemistry

For microscopy, freshly isolated liver pieces were fixed in a 10% formalin solution, embedded in paraffin, and 5-µm-thick sections were prepared. For the histological determination of hepatic steatosis, the sections were subjected to hematoxylin/eosin staining. For the assessment of senescence, we performed a hybrid histo-/immunochemical assay employing GL13 (commercially available as SenTraGor^®^), which is a lipophilic, biotin-linked Sudan Black-B analogue [45]. The evaluation was based on counting the number of GL13-positive hepatocytes per high-power field. A computerized Axioscan Z1 microscope (Zeiss, Oberkochen, Germany) or a DM LB microscope (Leica, Wetzlar, Germany) was used for picture acquisition. 

### 4.5. Determination of Telomere Length and Global DNA Methylation

For the determination of the telomere length and global DNA methylation in the isolated hepatic tissues, total DNA was firstly isolated from liver pieces using the commercially available DNeasy Blood & Tissue Kit (Qiagen, Hilden, Germany). Then, to quantitate the average telomere length in the hepatic tissue, a qPCR-based method using a commercially available kit was performed (Absolute Mouse Telomere Length Quantification qPCR Assay Kit, ScienceCell Research Laboratories, Carlsbad, CA, USA). Evaluation of the global DNA methylation of liver-derived DNA was performed by measuring the concentration of 5-methylcytosine (5-mC) using a commercially available kit (5-mC DNA ELISA Kit, Zymo Research, Irvine, CA, USA).

### 4.6. Measurement of Lipid Peroxidation in Liver

To assess the oxidative stress status of the isolated mouse livers, the levels of malondialdehyde (MDA), an end product of lipid peroxidation, were determined. To this end, a commercially available kit (Lipid Peroxidation (MDA) Assay kit, Abcam, Cambridge, UK) was used after homogenization of the tissue pieces in appropriate buffers provided by the kit.

### 4.7. Statistical Analysis

For all comparisons, a Mann–Whitney test was performed. The GraphPad Prism 8 software was used. Data are expressed as mean ± SEM and significance was set as *p*-value < 0.05.

## Data Availability

Data are available upon reasonable request.
